# New insights into the interplay between autophagy and cartilage degeneration in osteoarthritis

**DOI:** 10.3389/fcell.2022.1089668

**Published:** 2022-12-05

**Authors:** Xiaoman Lv, Ting Zhao, Youwu Dai, Mingqin Shi, Xiaoyi Huang, Yuanyuan Wei, Jiayan Shen, Xiaoyu Zhang, Zhaohu Xie, Qi Wang, Zhaofu Li, Dongdong Qin

**Affiliations:** ^1^ School of Basic Medical Sciences, Yunnan University of Chinese Medicine, Kunming, China; ^2^ The First School of Clinical Medicine, Yunnan University of Chinese Medicine, Kunming, China

**Keywords:** autophagy, osteoarthritis, mechanism, therapeutic applications, preclinical and clinical research

## Abstract

Autophagy is an intracellular degradation system that maintains the stable state of cell energy metabolism. Some recent findings have indicated that autophagy dysfunction is an important driving factor for the occurrence and development of osteoarthritis (OA). The decrease of autophagy leads to the accumulation of damaged organelles and macromolecules in chondrocytes, which affects the survival of chondrocytes and ultimately leads to OA. An appropriate level of autophagic activation may be a new method to prevent articular cartilage degeneration in OA. This minireview discussed the mechanism of autophagy and OA, key autophagy targets regulating OA progression, and evaluated therapeutic applications of drugs targeting autophagy in preclinical and clinical research. Some critical issues worth paying attention to were also raised to guide future research efforts.

## Introduction

Osteoarthritis (OA), the most common musculoskeletal disorder, is complex, multifaceted, and characterized by the degradation of articular cartilage and alterations in other joint tissues (Miyaki & Asahara, 2012). It is a highly prevalent disease, particularly in population over 65 years of age worldwide (Motta, Barone, Sica, & Selmi, 2022). Age is considered the strongest risk factor, injury, lifestyle, and congenital abnormality may further increase the risk of OA as well (Brumat et al., 2022). Cartilage degeneration is considered as the primary pathological change at the tissue level related to OA symptoms. The main pathogenesis of OA is the disorder of synthesis and degradation of articular cartilage and extracellular matrix (ECM). Articular cartilage is a kind of connective tissue composed of chondrocytes and ECM. Cartilage ECM is synthesized and secreted by chondrocytes and mainly consists of collagens (essentially type II) and aggregating proteoglycans (Eyre, 2002; Duan, Xie, & Liu, 2020). The abnormal expression of a set of aggrecanases is the main reason for cartilage degradation (e.g., a disintegrin and metalloproteinase with thrombospondin motifs ADAMTS-4 and -5) and collagenases (e.g., matrix metalloproteases, MMP-1, -3, -8, and -13), which are upregulated in OA (Charlier et al., 2016).

Studies have found that the degeneration of articular chondrocytes may be related to autophagy, which directly or indirectly affects the occurrence and development of OA (Takayama et al., 2014). Autophagy is an adaptive response, protecting cells from stress (Lamark & Johansen, 2012; Caramés, Olmer, Kiosses, & Lotz, 2015). During autophagy, parts of the cytoplasm and intracellular organelles are sequestered within characteristic double- or multi-membraned autophagic vacuoles and are finally delivered to lysosomes for bulk degradation (Almonte-Becerril et al., 2010; Yang & Klionsky, 2010). Three types of autophagy have been distinguished on the basis of the mechanism of cargo sequestration, including microautophagy, chaperone-mediated autophagy, and macroautophagy (Hansen, Rubinsztein, & Walker, 2018). Among them, macroautophagy, degradation of cytosolic material *via* sequestration into double-membrane vesicles called autophagosomes that subsequently fuse with lysosomes, is the most reported regarding the pathogenesis of OA (Jeon & Im, 2017). About 35 different conserved autophagy-related (ATG) genes encode proteins involved in the main steps of the macro-autophagy process (Parzych & Klionsky, 2014). Normal human cartilage can express high levels of autophagy regulators, including Unc-51-like kinase 1 (ULK1), beclin1, and LC3-II (Caramés, Taniguchi, Otsuki, Blanco, & Lotz, 2010; Zhang et al., 2015). The inhibition of autophagy caused OA-related gene expression changes in human chondrocytes, while the induction of autophagy prevented this (Sasaki et al., 2012). Targeted deletion of ATG in chondrocytes can promote cell death (Vuppalapati et al., 2015). Furthermore, the loss of proteoglycan is associated with reduced autophagic markers in OA (Kao et al., 2022). As essential organelles in chondrocytes, mitochondria supply energy and play vital roles in cellular metabolism and proliferation. Mitochondrial autophagy (also called mitophagy) is a specific type of autophagy that selectively removes damaged or depolarized mitochondria to maintain mitochondrial quality control (Yamashita & Kanki, 2017; Liu et al., 2022). Defective mitophagy leads to the accumulation of damaged mitochondria in the cytoplasm and cellular dysfunction (Lou et al., 2020). A study has found that mitophagy-related genes are highly expressed in OA patients’ cartilage (Shin et al., 2019). In addition, promoting mitophagy can protect the cartilage of OA patients (Jin et al., 2022). Therefore, insufficient autophagy can be associated with mitochondrial change in the pathogenesis of OA. Activating autophagy in degenerated cells may be a feasible and effective method to slow articular cartilage degeneration.

This minireview discussed the mechanism of autophagy and OA, key autophagy targets regulating OA progression, and evaluated therapeutic applications of drugs targeting chondrocyte autophagy in preclinical and clinical research. Some critical issues worth paying attention to were also raised to guide future research efforts.

## Role and potential mechanism of chondrocyte autophagy in osteoarthritis

**FIGURE 1:**
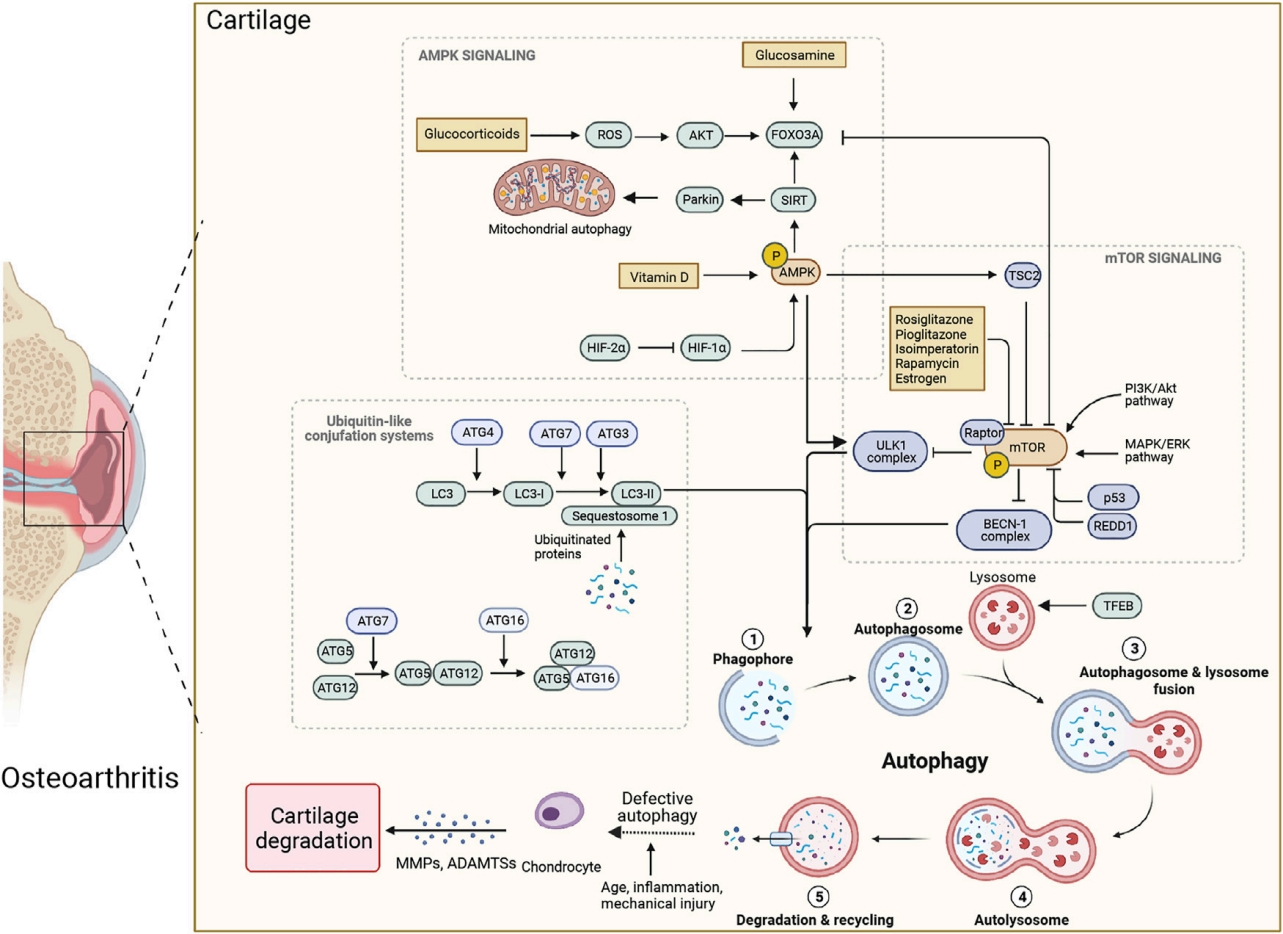
Therapeutic modulators of autophagy and associated mechanisms in osteoarthritis (OA). Autophagy is a multistep process that includes: ① phagophore formation; ② expansion elongation of phagophores to produce autophagosome; ③ autophagosomes fuse with endosomes and lysosomes; ④ autolysosome formation; ⑤ degradation of sequestered cargo in the autolysosome and recycling. Defective autophagy can lead to extracellular matrix degradation, resulting in OA. The mammalian target of rapamycin (mTOR) and AMP-activated kinase (AMPK) are the main regulators of chondrocyte autophagy in OA, with mTOR acting as an inhibitor and AMPK as an activator. The AMPK could phosphorylate and activate the sirtuin1 (SIRT1) and forkhead box class O 3a (FOXO3a), triggering autophagic flux through unc-51 like autophagy activating kinase 1 (ULK1), then suppressing mTOR. ULK1-mediated phosphorylation of AMPK constitutes a negative regulatory feedback loop. The mTOR can inhibit ULK1 and beclin-1 complexes, causing activation of autophagy. In addition, the transition from phagophore to autophagosome depends on the activity of two ubiquitin-like conjugation systems, the ATG5–ATG12 and LC3 system. The ubiquitin-like protein of ATG12 attaches to ATG5 with a covalent bond, and combines with ATG16 to elongate the pre-autophagosomal membrane. LC3-II is transformed from LC3-I through the lipidation of the ubiquitin-like reaction and binds to autophagic vesicles. Glucosamine can promote autophagy in cartilage through activating the dephosphorylation of FOXO3a. In contrast, glucocorticoids can stimulate FOXO3a and activate autophagy through a higher level of reactive oxygen species. Vitamin D can alleviate OA through AMPK-mTOR pathway. The rosiglitazone, pioglitazone, rapamycin, estrogen and isoimperatorin can inhibit mTOR activity, making them good candidates for potential therapies of OA.

Autophagy could promote either chondrocyte survival or death depending on the age, the presence and stage of OA, and the type of autophagy induction (Almonte-Becerril et al., 2010; Hwang, Yang, Park, & Kim, 2015). The pathogenesis of OA is mostly about aging (Terman, Kurz, Navratil, Arriaga, & Brunk, 2010; Bouderlique et al., 2016). The imbalance of catabolism and anabolism in the ECM is related to aging because the more vulnerable joint cannot afford damage from outside (Rahmati, Nalesso, Mobasheri, & Mozafari, 2017). Further, aging has a significant impact on autophagy-mediated chondrocyte survival. Studies have confirmed that autophagy-related proteins, such as Unc-51-like kinase 1 (ULK1), beclin-1, and LC3, were highly expressed in human chondrocyte clusters, whereas a reduced expression of these proteins was observed in the aged population (Caramés et al., 2010). Decrease of autophagy leads to the accumulation of intracellular damaged organelles and macromolecules, affecting chondrocyte survival, and ultimately leading to age-related OA (Bouderlique et al., 2016) (Figure 1). Aging may accelerate chondrocytes’ death by inhibiting chondrocytes’ autophagy, which promotes the development of OA.

In the early phase of OA, chondrocytes proliferate, giving rise to OA-typical clusters (Kim & Blanco, 2007). Autophagy is activated as an adaptive response to sublethal conditions, with the aim to avoid cell death (Klionsky, 2005; Levine & Yuan, 2005). Some studies have found that the ULK1, beclin-1, LC3 protein expression is decreased in the superficial zone, while these proteins are strongly expressed in the middle and deep zone (Kao et al., 2022). In later stages, there is an all-layered autophagic genetic change, including not only reduced ULK1, LC3, and beclin-1 but also fewer ATG3, ATG5, and ATG12. In later stage of OA, chondrocytes showed decreased autophagy and increased apoptosis. Apoptosis occurs when autophagy causes excessive consumption of intracellular proteins and organelles, which makes cells unable to survive. The reduction in key regulators of chondrocyte autophagy is a combination between classical apoptosis and autophagy (Almonte-Becerril et al., 2010). Autophagy is chondroprotective by regulating apoptosis, which is thought to be in balance with apoptosis when increased chondrocyte apoptosis occurs with lower expression of autophagy regulators in OA (Caramés et al., 2010).

In addition, autophagy can function to promote cell survival or cell death, depending on the type of cellular stress. Starvation and catabolic stress can increase autophagy in chondrocytes (Sasaki et al., 2012). However, autophagy related proteins (LC3-II, an autophagosomal marker in mammals) appear to decrease in mechanically injured cartilage (Caramés et al., 2012b; Vinatier, Domínguez, Guicheux, & Caramés, 2018). Additionally, mitochondrial dysfunction of chondrocytes showed an early increase in autophagy, which is a compensatory mechanism. However, when long-term pressure exceeds cell compensation, mitochondria in chondrocytes are damaged, leading to a massive increase in reactive oxygen species (ROS) and eventually OA (López de Figueroa, Lotz, Blanco, & Caramés, 2015). Therefore, autophagy has an essential role in protecting chondrocytes from different stressors. However, the relationship between autophagy and chondrocyte death has not been fully understood, and further research is needed to verify it.

## Key autophagy targets regulating osteoarthritis progression

Many molecules participate in the regulation of autophagic activity to affect the occurrence and development of OA, such as the mammalian target of rapamycin (mTOR), AMP-activated protein kinase (AMPK), non-coding RNA (ncRNA), *etc.* Studies have confirmed that the mTOR is the core target in regulating autophagy, which plays a vital role in cartilage growth and development and in altering articular cartilage homeostasis (Pal, Endisha, Zhang, & Kapoor, 2015). Furthermore, the specific knockdown of peroxisome proliferator activated receptor γ (PPARγ) can accelerate OA associated with aberrant mTOR signaling in the articular cartilage (Vasheghani et al., 2015). The inhibition of the PI3K/AKT/mTOR signaling pathway can significantly promote the autophagy level of OA chondrocytes (Chen, Crawford, & Xiao, 2013; Sun et al., 2020). In addition, tRNA-derived fragment tRF-5009A has been reported to have critical regulatory roles in OA, which regulates autophagy and cartilage degeneration by targeting mTOR (Deng et al., 2022). Regulated in development and DNA damage response 1 (REDD1), an endogenous mTOR inhibitor, was found to be decreased in OA cartilage (Charlier et al., 2016). It is highly expressed in normal human cartilage and reduced in OA patients (Alvarez-Garcia et al., 2016). Research has confirmed that REDD1 can regulate autophagy and mitochondrial biosynthesis to maintain the viability of chondrocytes (Alvarez-Garcia et al., 2017). REDD1 deficiency exacerbates the severity of injury-induced OA. Reduced REDD1 expression thus represents a novel mechanism for the defective autophagy observed in OA (Figure 1).

AMPK is a positive autophagy regulator, inhibiting mTOR from activating autophagy. After activation of AMPK, ULK1 and beclin1-VPS34 complex are phosphorylated and activated to promote the induction and formation of autophagosomes (Li & Chen, 2019). The increase of AMPK may be related to a change in the chondrocyte energy charge, which may promote autophagy. Dysfunction of AMPK activity has been associated with reduced autophagy, impaired mitochondrial function, excessive ROS generation, and inflammation in joint tissue, which leads to articular cartilage degeneration and synovial inflammation (Wang J. et al., 2020). Silent information regulator T1 (SIRT1) and forkhead box class O 3a (FOXO3a), as the signaling molecule downstream of AMPK, have been shown to trigger the formation of autophagosomes and activate autophagy by regulating the expression of ATG proteins (Hu et al., 2019; Wang J. et al., 2020). SIRT1 regulates autophagy by interacting with autophagy-related ATG7, which may become a more critical target in OA treatment (Liao et al., 2021). SIRT1-conditional knockout mice exhibit increased MMP13 and ADAMTS5 levels (Vinatier et al., 2018). SIRT3 can maintain the normal function of mitochondria and protect chondrocytes. The intra-articular SIRT3 overexpression alleviated the severity of OA-induced joint damage (Xu et al., 2021). Further, the FOXO may play a crucial role in postnatal cartilage development, maturation, and homeostasis and protects against OA-associated cartilage damage (Matsuzaki et al., 2018).

In addition, the putative kinase protein 1 (PINK1) is a serine kinase that can target mitochondria. Parkin is an E3 ubiquitin ligase that eliminates damaged mitochondria in OA chondrocytes (Ansari, Khan, Ahmad, & Haqqi, 2018). The PINK1-Parkin pathway can target to clear damaged mitochondria, reduce cell damage caused by oxidative stress, and improve chondrocyte survival rate (Wang et al., 2019). The transcription factor EB, a master regulator for autophagic flux, can alleviate articular cartilage degeneration and enhance autophagic activity (Zheng et al., 2018). Hypoxia-inducible factor-1α (HIF-1α) mediated mitophagy has a protective role in OA. The expression of HIF-1α was increased in human and mouse OA cartilage (Hu et al., 2020). HIF-1 inhibits mTOR signaling and ultimately enhances the autophagy activity of chondrocytes under hypoxia (Bohensky, Leshinsky, Srinivas, & Shapiro, 2010). Suppressing HIF-1α degradation can promote mitochondrial autophagy and alleviate cartilage degeneration (Hu et al., 2020). In contrast, HIF-2 is a potent negative regulator of autophagy in maturing chondrocytes, which promotes the degradation of chondrocyte ECM and is elevated in OA cartilage (Duan et al., 2020) (Figure 1).

For note, ncRNA has been confirmed to mediate autophagy in chondrocytes, such as microRNA, long non-coding RNA (lncRNA), and circular RNA (circRNA). Many microRNAs are commonly involved in the process of autophagy in OA (44–47). MiR-155 is an inhibitor in chondrocyte autophagy, which can alleviate key autophagy regulators contributing to the autophagy defects of OA (D'Adamo et al., 2016). MiR-9, MiR-34a, and miR-449 have been demonstrated to significantly reduce the expression of SIRT1 in chondrocytes (D'Adamo et al., 2017; Park et al., 2016; Yan et al., 2016). MiR-27a can enhance the autophagy and apoptosis of IL-1β-treated chondrocytes through PI3K/AKT/mTOR signaling (Cai et al., 2019). In addition, lncRNAs have been noticed to participate in OA through autophagy. LncRNA-GAS5 expresses highly in OA cartilage tissues, which is able to bind to miR-144 and regulate the expression of mTOR, inhibiting autophagy of OA chondrocytes (Ji, Qiao, Liu, & Wang, 2021). Hox transcript antisense intergenic RNA-induced apoptosis is mediated by sponging miR-130a-3p to repress chondrocyte autophagy in OA (He & Jiang, 2020). LncRNA-POU3F3 can inhibit chondrocytes, restraining autophagy and alleviating the pathogenesis of OA by regulating the miR-29a-3p/FOXO3 axis (Shi et al., 2022). Mesenchymal stem cell-derived exosome-mediated lncRNA KLF3-AS1 can repress autophagy of chondrocytes in OA (Wen, Lin, Zou, Lin, & Liu, 2022). Sex-determining region Y-box 4tbox4-activated lncRNA-MCM3AP antisense RNA 1 aggravated OA progression by modulating autophagy and ECM degradation *via* targeting miR-149-5p/Notch1 axis (Xu et al., 2022). Additionally, the circRNAs have also been involved in OA microenvironment for control of autophagy to perturb the situation of inflammation such as hsa_circ_0005567, ciRS-7, circPan3, has_circ_0037658, circMELK, and circRHOT1 (Zhang, Cheng, Rong, Tang, & Gui, 2020; Zhou et al., 2020; Sui, Liu, Que, Xu, & Hu, 2021; Zeng et al., 2021; Man et al., 2022; Zhang et al., 2022). Therefore, taking transcription factors, microRNA, lncRNA, circRNA, and autophagy inhibitors as entry points can serve as potential therapeutic targets for OA and represent a surprising new lead in the search for drugs to treat OA. However, more high-quality evidence is needed to confirm the appropriate therapeutic target of OA in clinical practice.

## Drugs targeting autophagy for osteoarthritis treatment

A variety of drugs regulating autophagic activity are used to treat OA. Rapamycin affected the mTOR signaling pathway in mouse knee joints as indicated by the inhibition of ribosomal protein S6 phosphorylation, a target of mTOR, and activation of LC3 (Caramés et al., 2012a). Rapamycin can prevent cell death and increase the expression of aggrecan and type II collagen while decreasing MMP-13 and ADAMTS5 in OA chondrocytes (Caramés et al., 2012a; Zhang et al., 2015). The intra-articular injection of rapamycin in a murine model could reduce vascular endothelial growth factor, collagen type X alpha 1, and MMP13 expression, leading to a delay in articular cartilage degradation (Takayama et al., 2014). However, long-term use of rapamycin may cause adverse events, such as headache, nausea, dizziness and epistaxis (Figure 1).

2-amino-2-deoxy-β-d-glucopyranose (glucosamine) is widely used to improve the symptoms and to delay the structural progression of OA (Conrozier & Lohse, 2022). Glucosamine is an effective autophagy activator, and autophagy enhancement mainly depends on the Akt/FOXO/mTOR pathway (Caramés et al., 2013). It has been found that the dual role of glucosamine in autophagy in human chondrocytes depends on exposure time (Kang et al., 2015). The exposure of glucosamine to chondrocytes activated autophagy, peroxidation, and pexophagy. However, long-time exposure of glucosamine may have the opposite effects due to the accumulation of peroxisomal dysfunction and long-chain fatty acids. Treatment of chondrocytes with glucosamine exerts exposure time-dependent dual effects on autophagy (Hochberg et al., 2012; Kang et al., 2015; Bruyère, Altman, & Reginster, 2016).

Glucocorticosteroid drugs have been used to treat early-stage OA, which may also lead to autophagy initiation. However, multiple administrations of glucocorticosteroid drugs may destroy the articular cartilage, induce human chondrocytes’ mitochondrial dysfunction, and increase ROS (Li, Chen, Li, Zhang, & Chen, 2022). In turn, dexamethasone may significantly attenuate the expression of MMP-13 in human OA chondrocytes through an mitogen-activated protein kinase phosphatase-1 and p38 mitogen-activated protein kinases-dependent manner (Lehtola et al., 2022). A study found that increased autophagy is an adaptive response to protect chondrocytes from short-term glucocorticosteroid exposure, whereas prolonged glucocorticosteroid drug treatment decreases autophagy and increases apoptosis (Liu, Wang, Zhao, Zhang, & Song, 2014). Thus, autophagy may be one of the essential mechanisms of glucocorticoids in the treatment of OA.

Many small molecule compounds and natural plant components play protective roles in OA by activating autophagy, such as isoimperatorin, delphinidin, celastrol, curcumin and astragaloside IV (Liu, Meng, Jing, & Zhou, 2017; Ouyang, Jiang, Fang, Cui, & Cai, 2017; Lee et al., 2020; Xiao et al., 2020; Dai et al., 2021). Baicalin protects chondrocytes against the degradation of ECM through activating autophagy *via* miR-766-3p/AIFM1 axis (Li, Cheng, & Liu, 2020). Dihydroartemisinin can suppress the levels of inflammatory factors in chondrocytes by promoting autophagy *via* inhibition of nuclear factor kappa-B pathway (Jiang et al., 2016). Trehalose can ameliorate endoplasmic reticulum stress and oxidative stress-mediated mitochondrial dysfunction *via* autophagic flux restoration and selective autophagy stimulation (Tang et al., 2017). Resveratrol can inhibit OA disease progression by activating SIRT1 (Deng et al., 2019).

In addition, the chemical autophagy inducer Torin 1 is an mTOR inhibitor, inhibiting the degenerative changes of experimental OA by activating autophagy (Cheng, Guo, & Meng, 2016). A study has demonstrated that fenofibrate, a PPARα agonist used for dyslipidaemias in humans, reduced both senescence and inflammation, increased autophagic flux, and increased autophagy in both ageing human and OA chondrocytes (Nogueira-Recalde et al., 2019). Metformin increased phosphorylated levels of AMPKα and upregulated SIRT1 protein expression, leading to an increase in autophagy, which may aid the development of novel therapeutic approaches for OA treatment (Wang C. et al., 2020). Parathyroid hormone-(1–34) can alleviate knee OA progression in rats *via* autophagy (Chen et al., 2018). Active vitamin D could be crucial in autophagosome aggregating and activates chondrocyte autophagy to reduce OA *via* mediating the AMPK-mTOR signaling (Kong et al., 2020). Estrogen may prevent articular cartilage destruction by promoting chondrocyte autophagy *via* the ERK-mammalian target of mTOR signaling (Ge et al., 2019). The PPARγ agonists rosiglitazone and pioglitazone also indirectly inhibit mTOR activity, making them good candidates for potential therapies in OA.

## Perspectives and conclusion

Autophagy dysfunction is an important driving factor for the occurrence and development of OA. Autophagy is a relatively balanced state under physiological conditions. When there is external stressor, the damaged organelles and the wrong proteins (such as drug stimulation, oxidative stress, diseases, *etc.*) are removed to improve the cell survival rate. Furthermore, when the cells are “weak,” they have no or deficient ability to autophagy. The cells are damaged by toxic substances, making survival difficult. An appropriate level of autophagic activation can effectively remove damaged organelles and macromolecules that need to be degraded to a certain extent, preventing chondrocyte damage and providing a good intracellular environment to promote the survival of chondrocytes. Given the critical role of autophagy in the pathogenesis of OA, the targeted autophagy pathway provides a new direction for the treatment of OA.

The relationship between chondrocyte autophagy targets autophagic activity in OA needs to be elucidated. Nevertheless, it means the likelihood of broadening the search for OA-related autophagy targets, which can serve as more comprehensive therapeutic targets or diagnostic biomarkers. Meanwhile, the research on drug regulation of OA autophagy focuses on animal research. Clinical studies are still dominated by single-center studies, lacking multicenter, large sample randomized controlled studies to evaluate drug efficacy. Therefore, further studies are needed to reduce sample heterogeneity and evaluate the effectiveness and safety of drugs in regulating autophagic signals. Nowadays, nanoplatforms combined to test therapeutic potential have become more common, which may have plenty of opportunities for the progress of autophagy-related treatment in OA. It is worth noting that the pathogenesis of OA is complex and is closely related to inflammation, aging, proliferation, and apoptosis. These pathogenesis have different autophagy needs in chondrocyte function and cell fate determination. Therefore, before the clinical application of autophagic active drugs to target the treatment of OA, it is necessary to conduct a comprehensive study on the relationship between crosstalk effects and multitargeting relationships.
